# Meta-Analysis of Expression Profiling Data Indicates Need for Combinatorial Biomarkers in Pediatric Ulcerative Colitis

**DOI:** 10.1155/2020/8279619

**Published:** 2020-01-29

**Authors:** Xinxiu Li, Eun Jung Lee, Danuta R. Gawel, Sandra Lilja, Samuel Schäfer, Huan Zhang, Mikael Benson

**Affiliations:** ^1^Centre for Personalized Medicine, Linköping University, Linköping, Sweden; ^2^Department of Otorhinolaryngology, Yonsei University College of Medicine, Seoul, Republic of Korea; ^3^Crown Princess Victoria Children's Hospital, Linköping University Hospital, Sweden

## Abstract

**Background:**

Unbiased studies using different genome-wide methods have identified a great number of candidate biomarkers for diagnosis and treatment response in pediatric ulcerative colitis (UC). However, clinical translation has been proven difficult. Here, we hypothesized that one reason could be differences between inflammatory responses in an inflamed gut and in peripheral blood cells.

**Methods:**

We performed meta-analysis of gene expression microarray data from intestinal biopsies and whole blood cells (WBC) from pediatric patients with UC and healthy controls in order to identify overlapping pathways, predicted upstream regulators, and potential biomarkers.

**Results:**

Analyses of profiling datasets from colonic biopsies showed good agreement between different studies regarding pathways and predicted upstream regulators. The most activated predicted upstream regulators included TNF, which is known to have a key pathogenic and therapeutic role in pediatric UC. Despite this, the expression levels of *TNF* were increased in neither colonic biopsies nor WBC. A potential explanation was increased expression of *TNFR2*, one of the membrane-bound receptors of TNF in the inflamed colon. Further analyses showed a similar pattern of complex relations between the expression levels of the regulators and their receptors. We also found limited overlap between pathways and predicted upstream regulators in colonic biopsies and WBC. An extended search including all differentially expressed genes that overlapped between colonic biopsies and WBC only resulted in identification of three potential biomarkers involved in the regulation of intestinal inflammation. However, two had been previously proposed in adult inflammatory bowel diseases (IBD), namely, MMP9 and PROK2.

**Conclusions:**

Our findings indicate that biomarker identification in pediatric UC is complicated by the involvement of multiple pathways, each of which includes many different types of genes in the blood or inflamed intestine. Therefore, further studies for identification of combinatorial biomarkers are warranted. Our study may provide candidate biomarkers for such studies.

## 1. Introduction

Pediatric ulcerative colitis (UC) is a serious inflammatory bowel disease (IBD) in childhood. The incidence of UC is between 0.5 and 4.3 per 100,000 individuals. Increasing prevalence and hospitalization rates have been reported [1–3]. Early diagnosis and monitoring of disease are important to prevent progression, but complicated by highly variable disease manifestations, which may not only affect the gastrointestinal tract but also the skin and eyes [4–6]. Therefore, there is a great need for reliable biomarkers for early diagnosis and disease monitoring [7–10].

However, despite decades of painstaking research, including different forms of genome-wide analyses, such biomarkers have proven difficult to find. Currently, diagnostics are therefore to a large extent based on general biomarkers for inflammation, like CRP, in combination with clinical history, physical examination, and different forms of imaging and pathological analyses.

An important reason for difficulties in finding biomarkers is indicated by expression profiling studies of whole blood cells (WBC) and colon biopsies, which have identified hundreds of disease-associated genes [11–14]. A similar complexity has been found in other pediatric diseases [15]. We recently described hundreds of differentially expressed genes (DEGs) only in peripheral CD4^+^T cells from adult patients with UC. Collectively, these DEGs could separate adult patients from healthy controls with high accuracy [16].

However, for clinical purposes, gene expression profiles in sorted cell types are not practical. Instead, such profiles could, ideally, be used to select a limited number of protein biomarkers that can be measured with routine methods, either in the blood or stools. From both basic and clinical perspectives, that selection should also be guided by an understanding of the relations between the biomarkers and pathogenic mechanisms in the inflamed intestine. To our knowledge, such relations have not been systematically investigated in pediatric UC. Here, we hypothesized that novel, and potentially more specific, biomarkers in the blood could be identified by searching for disease-associated pathways that overlap between cells in the peripheral blood and the inflamed colon. For this purpose, we performed meta-analyses of expression profiling data from WBC and colon biopsies from pediatric UC patients.

In summary, we found good agreement between pathogenic mechanisms indicated by meta-analyses of profiling studies of the colon from patients with pediatric UC. The most activated predicted upstream regulators included TNF, which is known to have a key pathogenic and therapeutic role in pediatric UC. Despite this, the expression levels of *TNF* were increased in neither colonic biopsies nor WBC. A potential explanation was increased expression of *TNFR2*, one of the membrane-bound receptors of TNF in the inflamed colon. We only found limited overlap between pathways and predicted upstream regulators in colonic biopsies and WBC. An extended search including all differentially expressed genes that overlapped between colonic biopsies and WBC only resulted in identification of three potential biomarkers involved in regulation of intestinal inflammation. However, two had been previously proposed in adult inflammatory bowel diseases (IBD), namely, MMP9 and PROK2 [17, 18]. Taken together, our analyses indicate complex relations between expression profiles in the peripheral blood and inflamed colon from patients with pediatric UC. Clinical implications of our study are that identification of reliable novel biomarkers may require simultaneous analyses of the peripheral blood and inflamed tissues in order to find combinations of biomarkers that reflect important disease mechanisms.

## 2. Materials and Methods

### 2.1. Data Collection Strategy

We performed a search for microarray expression profiling datasets to examine DEGs among pediatric patients with IBD. We searched NCBI Gene Expression Omnibus (GEO) with the following key words: “pediatric”, “child”, “children”, “IBD”, “inflammation bowel disease”, “UC”, and “ulcerative colitis”. The following inclusion criteria were used: (1) the samples were human and included both UC patients and healthy controls; (2) the age of patients should be less than 18; (3) all the datasets were accessible as raw or processed data. The following was extracted: (1) GEO accession, (2) array platform, (3) sample type, (4) and number of controls and patients. Studies not matching these selection criteria were excluded from the analyses (Figure S1). The analyses only included pediatric patients with UC and not patients with Crohn's disease.

### 2.2. Identification of DEGs

GEO2R was used to identify DEGs between pediatric UC patients with healthy controls [19, 20]. We used the Benjamini & Hochberg false discovery rate method to adjust for multiple comparisons. The adjusted *P* values (*q*-value) are listed in the *q*-value column of the results. The selection criterion for DEGs was a *q*-value less than 0.05. Please note that we consistently use italics to denote mRNAs, as opposed to when we refer to proteins. Also, human and mouse transcripts are written differently, for example, *TNFR1* (human) and *Tnfr1* (mouse).

### 2.3. Pathway and Upstream Regulators by Ingenuity Pathway Analysis

To identify canonical pathways and upstream regulators, we used the Ingenuity Pathway Analysis (IPA) based on DEGs from the different datasets [21]. Briefly, we performed the core analysis to identify significant canonical pathways that were enriched among the DEGs and predict significant activated upstream regulators [21]. For the pathways, *P* value < 0.05 and *z*‐score > 2 were considered statistically significant [21]. We mainly focused on seven types of molecules as upstream regulators, namely, cytokine, complex, group, growth factor, ligand-dependent nuclear receptor, G-protein coupled receptor, and transmembrane receptor.

### 2.4. Identification of Biomarkers and Enrichment Analysis

To predict biomarkers, we performed the biomarker filter and comparison analysis of IPA and mainly focused on extracellular biomarkers. The selection criteria for biomarkers were the following: we preselected proteins that were (a) DEGs; (b) upregulated or downregulated in UC compared to healthy (∣fold change∣ > 2 in colon datasets, ∣fold change∣ > 0.5 in blood datasets); and (c) predicted to be secreted by the Human Protein Atlas [22].

## 3. Results

### 3.1. Pediatric UC Eligible Microarray Datasets

We found, and included, three datasets from pediatric UC that matched our criteria (Table 1). Of the three datasets, two were from colonic biopsies, namely, GSE9686 [12] and GSE10616 [13] and one from WBC, GSE119600 [14]. These datasets are summarised in Table 1.

### 3.2. Identification of DEGs in mRNA Microarray Data from Colonic Biopsies from Patients with UC

**Figure 1:**
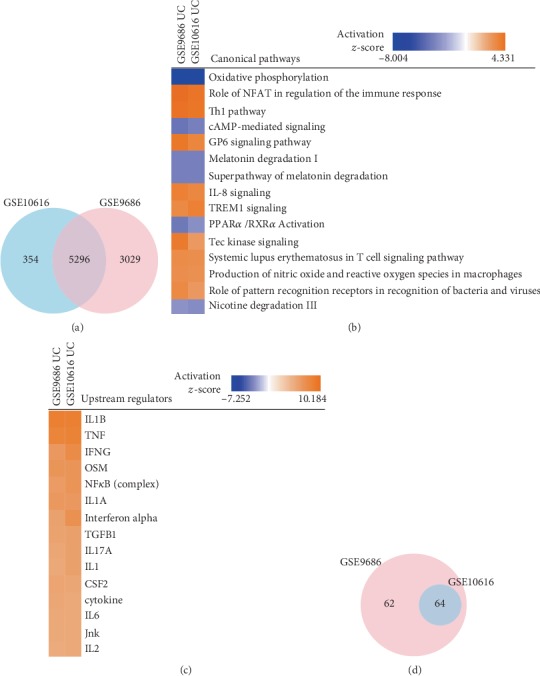
Analysis of shared pathways and upstream regulators in two expression profiling datasets of colonic biopsies from patients with ulcerative colitis. (a) Venn diagram of differentially expressed genes from the two datasets. (b) Canonical pathway comparisons between the two datasets. Orange indicates upregulated, and blue indicates downregulated pathways. Color intensity corresponds to significance, as indicated by the activation *z*-score bars below the pathways and upstream regulators. (c) Predicted upstream regulators of the mapped differentially expressed genes. (d) Venn diagram of predicted overlapping biomarkers from the two datasets. Dots mean nonsignificant values.

We found 5,296 DEGs that overlapped between the two datasets (Figure 1(a)). Interestingly, the five most significant DEGs were the same, namely, *SLC6A14*, *DUOX2*, *MMP1*, *MMP3*, and *MMP10*. Increased levels of MMPs have been previously described in inflammatory bowel diseases and their pathogenic relevance to UC supported by association with genetic variants [23, 24].

### 3.3. Identification of Pathways and Upstream Regulators in Pediatric UC

The large number of DEGs, and their participation in many different biological processes, necessitated a strategy to prioritise potential biomarkers that reflected the most important processes. We therefore systematically analysed the DEGs for pathways and predicted upstream regulators [16]. We identified seventeen pathways that overlapped between the two datasets (*z*‐score > 2 and *P* value < 0.05, Figure 1(b)). The ten most significant shared pathways are listed in Table 2.

Of these, Colorectal cancer metastasis signaling and LPS/IL-1-mediated inhibition of RXR function have been previously described in different pediatric IBD datasets [25, 26]. The Th1 pathway, TREM1 signaling, CD28 signaling in T helper cell, and Acute phase response signaling have been described in mixed datasets of pediatric and adult UC patients [27]. However, the remaining pathways have not been described in the pediatric UC.

Next, we analysed the two datasets to find upstream regulators that were predicted to be significantly upregulated in all two. This resulted in identification of 140 activated predicted regulators (*z*‐score > 2). The ten most significant regulators were all predicted to be upregulated, namely, IL1B, TNF, IFNG, OSM, NF*κ*B, IL1A, IFNA, TGFB1, IL17A, and IL1 (Figure 1(c)). These predictions agreed with previous studies implicating IL1A and IL1B, TNF, IFNG, NF*κ*B, CSF2, and TGFB in pediatric IBD [26, 27]. Also, OSM has been implicated in adult IBD patients [28–30]. It is important to note that the upstream regulators were predicted based on their known effects on downstream groups of genes. In other words, if a group of genes showed coordinated changes, potential upstream regulators of those changes were identified, based on previous experimental data accumulated in IPA [21]. However, the identified regulators may not necessarily be differentially expressed. We therefore checked if those regulators actually were differentially expressed in all of the two datasets. We found that only 45 (32.1%) were differentially expressed (Table 3). These 45 DEGs included some of the top ten predicted regulators, such as *IL1A*, *IL1B* and *IFNG*, but not *TNF* and *OSM*.

### 3.4. Increased Receptor Expression May Explain Why Predicted Upstream Regulators Are Not Differentially Expressed

**Figure 2:**
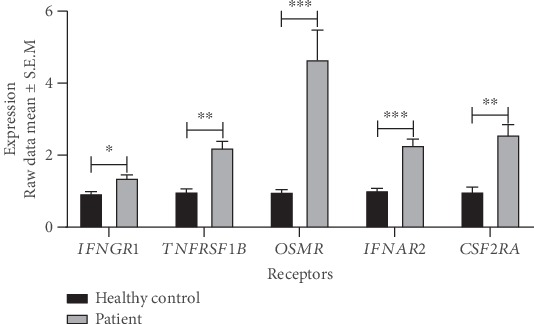
Expression levels of the receptors of predicted upstream regulators in pediatric ulcerative colitis. The expression levels are derived from the relative fluorescence of each transcript on the microarray chips. ^∗^*P* < 1 × 10^−3^, ^∗∗^*P* < 1 × 10^−4^, ^∗∗∗^*P* < 1 × 10^−5^. IFNGR1: interferon gamma receptor 1; TNFRSF1B: tumor necrosis factor receptor superfamily member 1B; OSMR: oncostatin M receptor; IFNAR2: interferon alpha and beta receptor subunit 2; CSF2RA: colony-stimulating factor 2 receptor alpha subunit.

Since 95 (67.9%) of the predicted 140 upstream regulators were not differentially expressed, we searched for explanations for this discrepancy. One explanation could be increased expression of the membrane-bound receptors of the regulators. Indeed, we found that 19 of the predicted 95 regulators were actually differentially expressed membrane receptors (Table S1). Those receptors included *TNFR2*, *OSMR*, *IFNAR2*, and *CSF2RA*, whose ligands were both known to be of pathogenic importance and predicted to be upregulated (Figure 2, Table S1).

Next, we investigated the expression levels of the receptors of the upstream regulators that were differentially expressed. Interestingly, those receptors showed a more complex pattern (Table S2). For example, *IFNGR1* showed increased expression (Figure 2), which is consistent with predicted increased activity of its ligand. However, *IFNGR2* decreased in all datasets. Similarly, the expression levels of *IL6R*, *IL17RB*, *IL17RD*, and *IL17RE* decreased in both datasets except *IL17RC* that decreased in one dataset (Table S2). Thus, the diagnostic interpretation of altered levels of upstream regulators that were ligands could be complicated by variable levels of the corresponding membrane-bound receptors. The clinical importance of this complication is further considered in the discussion, below.

### 3.5. Expanded Search for Candidate Biomarkers for Pediatric UC in Colonic Biopsies

Because of the potential difficulties in interpreting altered levels of soluble predicted upstream regulators, due to variable receptor expression, we expanded our search for biomarkers. Briefly, we searched for any DEG that encoded a possible biomarker based on our previously described criteria [16]. We focused on DEGs that encoded extracellular proteins that were likely to be measurable in stools or blood. We found 64 such DEGs that were shared between the two studies (Figure 1(d)). 59 were upregulated and five were downregulated. Some of these DEGs have been previously described as potential biomarkers or as having key pathogenic roles in the pathogenesis of UC or IBD. In summary, these included complement system: *C1QB*, *C1S*, *C3*, *C4A/C4B*, *C4BPA*, and *C4BPB*. These play an important role in the cellular immunity [31, 32]. C3 and C4B have previously been implicated in IBD [33, 34]; C-X-C chemokine family: *CXCL6*, *CXCL5*, *CXCL2*, *CXCL1*, *CXCL11*, *CXCL8*, *CXCL9*, *CXCL3*, *CXCL10*, *CXCL11*, and *CXCL13*; C-C motif chemokine ligand family: *CCL2*, *CCL11*, and *CCL18*; C-X-C chemokines: CXCL9 has been proposed as a drug target in UC [35]. C-C motif chemokines, like CCL11, have been found in high levels in the serum and biopsies of UC patients [36–38]. The MMP and TIMP families, like MMP1, MMP10, MMP12, MMP3, MMP7, MMP9, and TIMP1, play complex roles in IBD. On the one hand, they can have a protective role in maintaining the balance between extracellular matrix deposition and degradation. On the other hand, increased expression of MMPs may contribute to tissue damage in IBD [23, 24, 39]. Interestingly, we found a group of potential biomarkers that had not been described as biomarkers in UC, namely, *COL6A3*, *CTHRC1*, *EGFL6*, *FBN1*, *GLIPR1*, *GREM1*, *VCAN*, *MZB1*, and *PNLIPRP2* (Table 4).

Taken together, these and previous studies pointed to a large number of potential biomarkers for pediatric UC. To narrow down the number, we searched for genes that were also differentially expressed in WBC, as outlined below.

### 3.6. Identification of Genes, Pathways, Regulators, and Potential Biomarkers in WBC from Patients with UC

From a clinical perspective, measuring biomarkers in the blood may be more convenient than in stools. To find the former, we compared the profiling data from colonic biopsies with WBC to search for overlapping pathways and upstream regulators.

**Figure 3:**
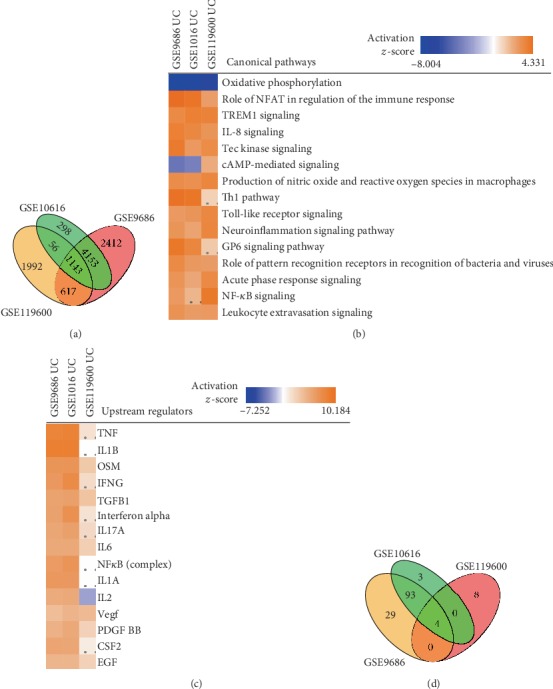
Ingenuity pathway analysis of expression profiling datasets of whole blood cells and colon biopsies from patients with ulcerative colitis. GSE119600 was the dataset of whole blood cells and GSE9686 and GSE10616 were datasets of colon biopsies. (a) Venn diagram of differentially expressed genes from the three datasets. (b) Canonical pathway comparisons between the three datasets. Orange indicates upregulated, and blue indicates downregulated pathways. Color intensity corresponds to significance. (c) Predicted upstream regulators of the mapped differentially expressed genes. (d) Venn diagram of predicted overlapped biomarkers from the three datasets. Dots mean a nonsignificant value.

Analysis of expression profiling data from WBC in pediatric UC identified 3,808 DEGs. There were 1,143 DEGs that overlapped between the three profiling studies of colonic biopsies and WBC (Figure 3(a)).

Pathway analysis identified 51 significantly activated pathways in WBC, but only eight that were significant in both WBC and colonic biopsies (*z*‐score > 2 and *P* value < 0.05, Table S3). This indicated that inflammatory mechanisms in the peripheral blood only partially reflected those in colonic tissue. However, the eight overlapping pathways had important pathogenic roles and therefore supported the potential of finding relevant biomarkers in the blood. The eight pathways were Tec kinase signaling, TREM1 signaling, IL-8 signaling, Production of nitric oxide and reactive oxygen species in macrophages, Leukocyte extravasation signaling, Neuroinflammation signaling pathway, Acute phase response signaling, and Role of pattern recognition receptors in recognition of bacteria and viruses (Figure 3(b) and Table S3). Of these, TREM1 signaling and Acute phase response signaling have been described as significantly activated pathways in both adult and pediatric UC [27]. Also, CRP, which is an important part of the acute phase response, is a clinically used biomarker for UC and many other diseases. The remaining six pathways have, however, not been described.

We found 21 upstream regulators whose predicted activity increased significantly in WBC. Of these, one, *OSM*, was a DEG (Figure 3(c) and Table S4). Taken together with increased expression of *OSMR* in colonic biopsies, this supports the potential of OSM as a biomarker. The limited overlap between pathways, upstream regulators, and potential biomarkers (Figures 3(b)–3(d) and Table S3) between WBC and colon biopsies led us to examine if predicted upstream regulators in colonic biopsies were differentially expressed in WBC. If so, those regulators could also be suitable to be measured as biomarkers in the blood because they could potentially regulate key pathways in the inflamed colon. We found that 13 of the predicted regulators in the colon were differentially expressed in WBC, namely, *TNF*, *OSM*, *IFNG*, *EDN1*, *IFNL1*, *CCL5*, *IL32*, *TNFSF12*, *BCR*, *CCL3*, *VEGFA*, *ITGAM*, and *LTA* (Table 5).

All the 13 regulators had predicted increased activity in the colon. Thus, to be suitable as biomarkers in the blood, their expression levels in the blood should also be increased. However, most of the 13 genes showed lower expression in patients than in controls, except for *OSM*, *EDN1*, *BCR*, *VEGFA*, and *ITGAM*. Of these five, only *OSM* and *ITGAM* showed expression fold changes of sufficient magnitude to be candidate biomarkers (∣logFC∣ > 0.4) (Table 5 and Materials and Methods).

This led us to search for potential biomarkers among all the DEGs in WBC, using the same criteria as described above for colonic biopsies. This resulted in identification of *FGFBP2*, *IL32*, *MUC6*, *LGALS3*, *PI16*, *ADM*, *PROK2*, *COL18A1*, *F5*, *JCHAIN*, *MZB1*, and *MMP9* (Table S5). Of these, all except, *FGFBP2*, *IL32*, *MUC6*, *LGALS3*, and *PI16* showed increased expression. We finally searched for biomarkers that overlapped with those identified in colonic biopsies and only found three, namely, MMP9, MZB1, and PROK2. Of these, MMP9 and PROK2 have been previously described as the potential biomarkers in adult IBD [17, 40, 41].

## 4. Discussion

Pediatric UC is a chronic inflammatory bowel disease that can lead to severe derangements in the growth, nutritional status, and psychosocial development of affected children. Despite the increasing understanding of the pathogenic mechanisms, which have led to new therapies that specifically targets those mechanisms, there is still a need for biomarkers for early diagnosis, stratification of patients, and disease monitoring. Here, we aimed to find reasons for the difficulties in identifying such biomarkers, as well as new candidate biomarkers.

We reasoned that proteins in the blood or stools would be most suitable for clinical use. Because of the molecular complexity of pediatric UC, we started by a meta-analysis of expression profiling data from colonic biopsies. We also reasoned that the selection of potential biomarkers should be guided by an understanding of pathogenic mechanisms, as indicated by pathways and upstream regulators.

The meta-analysis of profiling studies of the colon from patients with pediatric UC showed good agreement between different studies regarding pathways and upstream regulators. As a specific example, the expression of TNF-induced genes increased in all studies. This is consistent with the known pathogenic and therapeutic importance of TNF in pediatric UC. Therefore, measurement of TNF proteins in the blood or stools could be diagnostically important. However, while increased levels of TNF and other pro-inflammatory cytokines have been described in adult UC [42], none has become clinically accepted as biomarkers. Our analyses identified potential explanations. First, we did not find any local increase of *TNF* expression in the colonic biopsies. Second, the expression levels of *TNF* even decreased in WBC from the peripheral blood. Thus, these findings did not support measurement of TNF in the blood or stools for diagnostic purposes. Interestingly, the expression levels of many other predicted upstream regulators also did not increase in the colonic biopsies. This led us to analyse the expression levels of the membrane-bound receptors of TNF, as well as of other predicted regulators. We found that *TNFR2*, but not *TNFR1*, increased in patients. These findings are consistent with the two receptors having opposite roles in a mouse model of colitis [43]. *Tnfr1* ablation led to exacerbation of signs of colitis, including more weight loss, increased mortality, colon shortening and oedema, severe intestinal damage, and higher levels of myeloperoxidase compared to wild-type counterparts. By contrast, *Tnfr2* deficiency had the opposite effects. We also found increased expression of many other receptors of predicted upstream regulators that did not increase. This suggests that altered receptor expression can explain increased activity of TNF and other important regulators in pediatric UC. Another explanation could be changed levels of soluble receptors for the regulators. Indeed, soluble TNF receptors have been shown to correlate with disease activity in adult IBD [44]. As previously discussed by us and others, such receptors may have both inhibitory and activating roles, depending on their mechanisms of action, as well as their levels relative to their ligand and membrane-bound receptors [45]. Further analyses showed complex relations between the expression levels of the other predicted regulators and their membrane-bound receptors in colonic biopsies from the patients with pediatric IBD. For example, *IFNAR1* increased, while *IFNAR2* decreased in colonic biopsies. This is consistent with genome-wide association studies that have implicated the locus containing *IFNAR1* as a genetic risk factor for developing IBD [46].

Thus, diagnostic analyses of any predicted regulator in the blood or stools may need to take into account the relative expression levels of the corresponding levels of the cognate-soluble and membrane-bound receptors. This led us to extend our search for potential biomarkers to any gene that was differentially expressed in both the colon and WBC and also encoded an extracellular protein. In summary, we found that those genes only partially overlapped between colonic biopsies and WBC. However, we did identify three candidate biomarkers that did overlap, namely, MMP9, MZB1, and PROK2. Their pathogenic and diagnostic relevance is supported by involvement in the intestinal blood flow, leukocyte migration, and tissue degradation [39, 47–49]. Indeed, MMP9 and PROK2 have been proposed as diagnostic markers in adult IBD [17, 18]. Taken together with our findings, this suggests that future studies are warranted for MMP9, MZB1, and PROK2 as candidate biomarkers in pediatric UC.

Limitations of our study include that mRNA expression levels may not correlate with protein levels. However, we have previously found that profiling data can be exploited to identify potential protein biomarkers in adult IBD and other inflammatory diseases [16, 50, 51]. Other limitations are that the bioinformatics analyses of pathways and upstream regulators were based on a manually curated aggregate of multiple data sources, which may be confounded by, for example, cell type- or tissue-specific variations. On the other hand, the reliability of the findings is supported by agreement with known mechanisms and between the expression profiling data from two different datasets.

In summary, our findings indicate that the difficulties in finding specific biomarkers for pediatric UC depend on the complex underlying mechanisms, which include multiple pathways and regulators, each of which may be subdivided into multiple components such as ligands, soluble, and membrane-bound receptors. Moreover, those mechanisms may vary between different disease-associated compartments. Therefore, in order to select reliable biomarkers, studies that simultaneously analyse multiple mechanisms in the peripheral blood and inflamed tissues may be required. Our data may contribute to prioritisation of pathways and regulators for such studies.

## Figures and Tables

**Table 1:** Summary of the three genome-wide gene expression datasets that were used in this study.

GSE number	Samples	Source types	Platform
GSE9686 [12]	5 UC patients and 8 healthy controls	Colon biopsy	GPL5760 Affymetrix GeneChip Human Genome U133 Plus 2.0 Array
GSE10616 [13]	10 UC patients and 11 healthy controls	Colon biopsy	GPL5760 Affymetrix GeneChip Human Genome U133 Plus 2.0 Array
GSE119600 [14]	48 UC patients and 47 healthy controls	Whole blood	GPL10558 Illumina Human HT-12 V4.0 expression beadchip

**Table 2:** Pathways that overlapped between expression profiling data from two studies of colon biopsies of pediatric ulcerative colitis (*z*‐score > 2 and *P* value < 0.05).

Positive canonical pathway	Activation *z*-score		*P* value	
	GSE9686	GSE10616	GSE9686	GSE10616
Th1 pathway	4.160	4.012	6.76E-04	1.20E-04
GP6 signaling pathway^∗^	3.969	3.429	1.78E-03	4.90E-03
Tec kinase signaling	3.810	2.714	2.34E-03	1.29E-02
IL-8 signaling^∗^	3.666	3.349	6.17E-03	1.17E-02
Colorectal cancer metastasis signaling	3.334	2.333	1.66E-03	5.13E-04
TREM1 signaling	3.317	3.651	3.02E-05	3.24E-03
Role of pattern recognition receptors in recognition of bacteria and viruses^∗^	3.280	2.746	2.29E-02	1.29E-02
Systemic lupus erythematosus in the T cell signaling pathway	3.200	3.118	3.98E-03	2.69E-03
Production of nitric oxide and reactive oxygen species in macrophages^∗^	3.130	3.048	1.23E-02	3.02E-03
PKC*θ* signaling in T lymphocytes^∗^	3.046	2.771	8.13E-03	9.33E-04
Leukocyte extravasation signaling	2.951	2.630	3.72E-07	1.82E-07
Acute phase response signaling	2.898	2.263	6.92E-03	1.38E-03
Neuroinflammation signaling pathway^∗^	2.866	2.360	9.12E-03	1.15E-03
Osteoarthritis pathway^∗^	2.862	3.022	9.33E-04	1.82E-05
CD28 signaling in T helper cells	2.654	2.271	4.57E-02	2.24E-03
LPS/IL-1-mediated inhibition of RXR function	2.592	2.466	1.45E-05	1.23E-06
Chondroitin and dermatan biosynthesis^∗^	2.236	2.236	3.39E-02	6.76E-03

∗ indicates a pathway that has not been described in pediatric UC. GP6: glycoprotein VI; IL-8: interleukin-8; TREM1: triggering receptor expressed on myeloid cell-1; PKC*θ*: protein kinase C theta; LPS: lipopolysaccharides; IL-1: interleukin-1; RXR: retinoid X receptor.

**Table 3:** List of differentially expressed predicted upstream regulators (excluding receptors).

Gene symbol	Log FC		Activation *z*-score	
	GSE9686	GSE10616	GSE9686	GSE10616
*IL1B*	2.471	2.167	8.740	8.695
*IL1A*	2.144	1.794	6.531	6.501
*IFNG*	1.882	1.499	6.401	7.570
*TGFB1*	1.106	0.821	5.649	5.841
*IL17A*	0.527	0.411	5.390	5.877
*IL6*	2.407	1.970	5.218	5.250
*F3*	1.659	1.392	4.167	4.082
*EDN1*	-1.789	-1.526	4.120	4.969
*CXCL12*	1.624	0.916	4.042	4.590
*TGFB3*	1.375	0.943	3.806	3.694
*TYROBP*	1.941	1.479	3.582	3.138
*SELP*	1.499	1.462	3.484	3.194
*CSF1*	-0.484	-0.289	3.431	3.514
*TREM1*	1.325	1.291	3.356	3.195
*FGF2*	0.662	0.480	3.263	3.827
*NAMPT*	1.331	1.048	3.115	3.115
*CCL11*	3.210	2.610	3.068	3.164
*WNT5A*	3.419	2.573	3.031	2.883
*IL36B*	-0.370	-0.301	2.957	2.803
*PF4*	1.140	1.217	2.950	3.270
*TNFSF13B*	2.349	1.714	2.758	2.661
*TNFSF11*	2.151	1.625	2.654	3.091
*IFNW1*	-0.585	-0.458	2.639	2.442
*HGF*	0.758	0.748	2.619	3.894
*TNFSF15*	1.055	0.782	2.568	2.568
*IL33*	2.022	1.671	2.562	2.300
*ITGAM*	1.752	1.296	2.414	2.200
*CXCL8*	3.365	3.204	2.393	2.781
*CCL2*	3.748	2.672	2.376	2.471
*GDNF*	-0.312	-0.340	2.357	2.327
*IL3*	-0.570	-0.340	2.320	2.174
*AGT*	3.578	2.725	2.219	2.979

IL1B: interleukin 1B; IFNG: interferon gamma; IL1A: interleukin 1A; TGFB1: transforming growth factor beta 1; IL17A: interleukin 17A; IL6: interleukin 6; EDN1: endothelin 1; CXCL12: C-X-C motif chemokine ligand 12; TGFB3: transforming growth factor beta 3; FGF2: fibroblast growth factor 2; CSF1: colony-stimulating factor 1; TYROBP: TYRO protein tyrosine kinase binding protein; SELP: selectin P; TREM1: triggering receptor expressed on myeloid cell-1; HGF: hepatocyte growth factor; CCL11: C-C motif chemokine 11; NAMPT: nicotinamide phosphoribosyltransferase; PF4: platelet factor 4; WNT5A: Wnt family member 5A; IL36B: interleukin 36B; TNFSF11: TNF superfamily member 11; TNFSF13B: TNF superfamily member 13b; AGT: angiotensinogen; CXCL8: C-X-C motif chemokine ligand 8; TNFSF15: TNF superfamily member 15; IFNW1: interferon omega 1; IL33: interleukin 33; CCL2: C-C motif chemokine ligand 2; GDNF: glial cell-derived neurotrophic factor; ITGAM: integrin subunit alpha M; IL3: interleukin 3.

**Table 4:** Potential new biomarkers in UC.

Gene symbol	NCBI accession	GSE9686 (log FC)	GSE10616 (log FC)
*GREM1*	NM_013372	4.176	3.559
*CTHRC1*	NM_138455	3.952	3.116
*GLIPR1*	NM_006851	3.702	2.547
*COL6A3*	NM_004369	2.934	2.310
*EGFL6*	NM_015507	2.912	2.318
*VCAN*	NM_004385	2.891	2.303
*MZB1*	NM_016459	2.550	2.133
*FBN1*	NM_000138	2.445	2.016
*PNLIPRP2*	NM_005396	-2.305	-2.046

GREM1: gremlin 1; CTHRC1: collagen triple-helix repeat containing 1; GLIPR1: glioma pathogenesis-related protein 1; COL6A3: collagen type VI alpha 3 chain; EGFL6: EGF-like domain multiple 6; VCAN: versican; MZB1: marginal zone B and B1 cell-specific protein; FBN1: fibrillin-1; PNLIPRP2: pancreatic lipase-related protein 2.

**Table 5:** The expression level of genes in whole blood cells, which were predicted to be upstream regulators in colonic biopsies.

Gene symbol	Activated *z*-score		GSE119600	
	GSE9686	GSE10616	LogFC	*q*-value
*TNF*	8.241	8.388	-0.476	9.04E-07
*OSM*	6.742	6.823	0.406	7.77E-03
*IFNG*	6.401	7.570	-0.447	7.26E-04
*EDN1*	4.120	4.969	0.076	3.00E-02
*IFNL1*	3.694	4.987	-0.152	8.12E-04
*CCL5*	3.079	2.776	-0.492	3.72E-03
*IL32*	2.937	2.753	-0.606	1.17E-05
*TNFSF12*	2.867	2.867	-0.175	1.20E-02
*BCR*	2.696	2.366	0.108	4.14E-02
*CCL3*	2.663	2.663	-0.230	2.29E-03
*VEGFA*	2.594	3.645	0.151	3.16E-04
*ITGAM*	2.414	2.200	0.597	6.17E-05
*LTA*	2.137	2.813	-0.235	2.23E-02

TNF: tumor necrosis factor; OSM: oncostatin M; IFNG: interferon gamma; EDN1: endothelin 1; IFNL1: interferon lambda 1; CCL5: C-C motif chemokine ligand 5; IL32: interleukin 32; TNFSF12: TNF superfamily member 12; BCR: breakpoint cluster region; CCL3: C-C motif chemokine ligand 3; VEGFA: vascular endothelial growth factor A; ITGAM: integrin alpha M; LTA: lymphotoxin alpha.

## Data Availability

The raw data supporting this meta-analysis are from previously reported studies and data, which have been cited. The processed data are included within the article. The full processed data in detail are also available from the corresponding author upon request.

## Supplementary Materials

Supplementary Materials Table S1: expression levels of receptors for predicted upstream regulators that were not differentially expressed. Table S2: expression levels of receptors to predicted upstream regulators that were differentially expressed. Table S3: pathways that overlapped between expression profiling data from three studies of colonic biopsies and whole blood cells from pediatric ulcerative colitis (*z*‐score > 2 and *P* value < 0.05). Table S4: list of differentially expressed predicted upstream regulators in whole blood cells Table S5: list of potential biomarkers for ulcerative colitis in whole blood cells. Figure S1: summary of workflow used for the microarray meta-analysis. Figure S2: volcano plots for three datasets. Volcano plots showing differentially expressed genes shared by all datasets. The red horizontal line denotes adjusted *P* value cutoff of 0.05.
