# Supplementary material for: Meta-Analysis of Expression Profiling Data Indicates Need for Combinatorial Biomarkers in Pediatric Ulcerative Colitis
Source: J Immunol Res. 2020 Jan 29;2020:8279619. doi: 10.1155/2020/8279619 (PMC7204128; doi:10.1155/2020/8279619)
Supplement: Supplementary Materials — Table S1: expression levels of receptors for predicted upstream regulators that were not differentially expressed. Table S2: expression levels of receptors to predicted upstream regulators that were differentially expressed. Table S3: pathways that overlapped between expression profiling data from three studies of colonic biopsies and whole blood cells from pediatric ulcerative colitis (z‐score > 2 and P value < 0.05). Table S4: list of differentially expressed predicted upstream regulators in whole blood cells Table S5: list of potential biomarkers for ulcerative colitis in whole blood cells. Figure S1: summary of workflow used for the microarray meta-analysis. Figure S2: volcano plots for three datasets. Volcano plots showing differentially expressed genes shared by all datasets. The red horizontal line denotes adjusted P value cutoff of 0.05. [file 8279619.f1.pdf]

Supplementary Data

Meta-analysis of expression profiling data indicates need for combinatorial biomarkers in pediatric ulcerative colitis

Xinxiu Li[1*], Eun Jung Lee[1,2]*, Danuta R. Gawel[1], Sandra Lilja[1], Samuel Schäfer[1], Huan Zhang[1,3], Mikael Benson[1,3]

[1]Centre for Personalized Medicine, Linköping University, Linköping, Sweden.

[2]Department of Otorhinolaryngology, Yonsei University College of Medicine, Seoul, Korea.

[3]Crownprincess Victoria Children's Hospital, Linköping University Hospital, Sweden

* Xinxiu Li and Eun Jung Lee contributed equally to this work, shared first authors

Correspondence should be addressed to Mikael Benson; mikael.benson@liu.se

Table S1. Expression levels of receptors for predicted upstream regulators that were not differentially expressed

The Upstream regulators(UR)	The receptors of UR	NCBI Accession	GSE9686		GSE10616	
			q-value	logFC	q-value	logFC
TNF	*TNFR1*	NM_001065	0.964449	-0.008	0.802000	-0.03
	*TNFR2**	NM_001066	0.000567	1.308	0.000056	1.32
OSM	*OSMR**	NM_003999	0.000004	2.28	0.000256	2.19
OSM/EBI3/ IL-27B	*gp130/IL6ST**	NM_175767	0.004510	1.24	0.002669	1.64
		NM_002184	0.004750	1.232	0.002640	1.64
CSF2	*CSF2RA**	NM_172248	0.000993	1.418	0.000547	1.45
		NM_172246	0.001640	1.67	0.000642	1.79
		NM_006140	0.001820	1.672	0.000605	1.86
		NM_172247	0.001820	1.742	0.000638	1.87
		NM_172245	0.001780	1.674	0.000524	1.89
		NM_172249	0.000954	1.249	0.001865	1.11
VEGF/VEGFA	*VEGFR3/FLT4*	NM_002020	0.135000	0.21	0.191192	0.25
		NM_182925	0.066300	-0.181	0.139729	-0.18
	*VEGFR2/FLK1/KDR**	NM_002253	0.000100	1.503	0.000453	1.88
IL1	*IL1R1*	NM_000877	0.003790	0.697	0.014902	0.64
	IL1R2	NM_004633	0.611000	-0.208	0.925082	0.05
		NM_173343	0.616000	-0.205	0.926827	0.05
IL18	*IL18R1**	NM_003855	0.000286	1.122	0.002777	1.12
		NM_003853	0.005910	0.86	0.048610	0.71
PDGF BB/PDGF (complex)	*PDGFRA**	NM_006206	0.021700	1.634	0.018271	2.02
		NM_002609	0.002460	1.128	0.048746	0.87
	PDGFRB	NM_033016	0.106000	-0.246	0.008526	-0.52
		NM_002608	0.150000	-0.24	0.048746	0.87
IL5	*IL5RA*	NM_175728	0.297000	0.123	0.258163	0.14
		NM_175725	0.318000	0.118	0.262857	0.14
		NM_000564	0.532000	0.063	0.423365	0.09
		NM_175726	0.530000	0.065	0.491669	0.08
		NM_175727	0.926000	0.009	0.851207	-0.02
		NM_175724	0.990000	0.001	0.876582	-0.02
IL7/TSLP	*IL7R*	NM_002185	0.013300	1.534	0.053356	1.61
IL12 (complex)	*IL12RB1*	NM_005535	0.997000	-4E-04	0.724499	-0.06
		NM_153701	0.997000	-4E-04	0.000684	1.5
	*IL12RB2**	NM_001559	0.008480	0.368	0.037771	0.35
IFN/IFNA1/IFN A2/IFNA13/IFNB1 /IFNE/IFNK	*IFNAR1*	NM_000629	0.019900	0.519	0.001944	0.72
	*IFNAR2**	NM_207585	0.000651	0.666	0.001634	0.75
		NM_207584	0.000018	1.501	0.000164	1.52
		NM_000874	0.000018	1.504	0.000168	1.52
CG	*LHCGR*	NM_000233	0.246000	-0.123	0.074687	-0.21
IL21	*IL21R**	NM_021798	0.000114	1.297	0.002189	1.47
		NM_181079	0.000119	1.299	0.002204	1.47
		NM_181078	0.000123	1.299	0.002223	1.47
EGF/BTC	*EGFR*	NM_201284	0.034400	-0.262	0.060171	-0.29
		NM_005228	0.007750	-0.402	0.054154	-0.36
		NM_201282	0.017100	-0.455	0.003126	-0.54
		NM_201283	0.023400	-0.306	0.032851	-0.36
CCL5/CCL3	*CCR1**	NM_001295	0.000850	2.092	0.000718	2.42
	CCR3	NM_001837	0.627000	-0.191	0.885801	-0.06
		NM_178329	0.585000	-0.213	0.857326	-0.08

Fibrinogen	*ITGB1* *	NM_002211	0.007160	0.684	0.001157	1.18
		NM_033668	0.188000	0.21	0.014916	0.46
		NM_033666	0.505000	0.053	0.432250	0.08
	ITGA5	NM_002205	0.086000	0.322	0.548843	0.16
	ITGA2B	NM_000419	0.421000	-0.075	0.055396	-0.24
IL17F	*IL-17RC*	NM_032732	0.013300	-0.288	0.009987	-0.42
		NM_153461	0.011800	-0.295	0.011586	-0.42
		NM_153460	0.014400	-0.285	0.010955	-0.42
IL17F/IL17C	*IL-17RA*	NM_014339	0.528000	0.064	0.614741	-0.06
SAA	*CD36*	NM_001001547	0.938000	0.05	0.698807	0.28
		NM_000072	0.938000	0.05	0.706475	0.27
		NM_001001548	0.376000	0.518	0.068235	1.25
LTA/LT	*LTBR*	NM_002342	0.007910	-0.34	0.090781	-0.25
IL27/EBI3	*IL27RA* *	NM_004843	0.000514	1.029	0.004754	1.14
IFNL1	*IL10RB*	NM_000628	0.009250	-0.409	0.077122	-0.34
	IFNLR1	NM_170743	0.009320	-0.483	0.055396	-0.46
Ap1	*TACI /BCMA/TNFRSF13B*	NM_012452	0.336000	0.181	0.198392	0.28
IL22	*IL22RA1*	NM_021258	0.270000	-0.3	0.687153	-0.14
	IL22RA2	NM_181310	0.018900	1.078	0.118424	0.78
		NM_181309	0.019000	1.077	0.117328	0.78
		NM_052962	0.019300	1.069	0.115673	0.78
IL23	*IL23R*	NM_144701	0.010100	-0.6	0.079804	-0.45
CCL3	*CCR4*	NM_005508	0.441000	-0.091	0.900976	-0.02
IL36A	*IL1RL2*	NM_003854	0.003870	-0.439	0.003214	-0.51
	IL1RAP *	NM_002182	0.048500	0.283	0.087739	0.33
		NM_134470	0.008990	0.415	0.009676	0.48
C5	*C5AR1* *	NM_001736	0.000735	0.961	0.019548	0.78
TGFB2	*TGFBR1* *	NM_004612	0.001150	1.164	0.001091	1.38
	TGFBR2 *	NM_001024847	0.003400	0.713	0.003924	0.9
		NM_003242	0.003470	0.712	0.004039	0.9
TSLP	*CRLF2*	NM_022148	0.365000	-0.13	0.296690	-0.17
IL17C	*IL-17RE*	NM_153483	0.000649	-0.656	0.003525	-0.7
		NM_153482	0.000687	-0.654	0.003553	-0.7
		NM_153481	0.000667	-0.655	0.003468	-0.71
		NM_153480	0.000709	-0.65	0.003707	-0.7
		NM_144640	0.000800	-0.643	0.005108	-0.68
MIF	*CXCR2* *	NM_001557	0.000031	2.744	0.001082	2.32
	CXCR4	NM_001008540	0.121000	1.096	0.207240	1.13
		NM_003467	0.121000	1.097	0.206988	1.13
	CD74 *	NM_001025159	0.000539	1.64	0.000350	2.17
		NM_004355	0.000172	1.891	0.000345	2.29
		NM_001025158	0.000172	1.892	0.000345	2.29
AP-1	*CTLA4*	NM_005214	0.007710	1.078	0.063021	0.94
		NM_001037631	0.007280	1.136	0.065263	0.98
PRL	*PRLR*	NM_000949	0.052300	-0.172	0.094922	-0.2
AIMP1	*FCER2*	NM_002002	0.884000	0.028	0.919485	-0.03
IGF2	*IGF2R*	NM_000876	0.792000	-0.034	0.962755	-0.01
GH1	*GHR*	NM_000163	0.238000	0.673	0.053277	1.08
LDL	*LDLR*	NM_000527	0.649000	0.167	0.540602	-0.22

* Positive logFC and q-value < 0.05

Table S2. Expression levels of receptors to predicted upstream regulators that were differentially expressed

The Upstream regulators(UR)	The receptors of UR	NCBI Accession	GSE9686		GSE10616	
			q-value	logFC	q-value	logFC
IL1B/IL1A	*IL1R1*	NM_000877	0.01490168	0.638078	0.05192	0.5047544
	IL1R2	NM_004633	0.92508222	0.04612376	0.73942	-0.1604044
		NM_173343	0.92682704	0.04514464	0.74207	-0.1588029
IFNG	*IFNGR1**	NM_000416	0.00203069	0.64796339	0.03232	0.4309898
	IFNGR2	NM_005534	0.02734678	-0.35199119	0.01784	-0.3421646
TGFB1	*TGFBR1*	NM_032811	0.07843288	-0.39924435	0.22272	-0.2552363
	TGFBR4	NM_030900	0.00012386	-1.21207349	0.00818	-0.9596834
		NM_199122	0.00012923	-1.20612685	0.00804	-0.957314
		NM_004749	0.00014338	-1.19615166	0.00813	-0.9598502
IL17A	*IL17RA*	NM_014339	0.61474102	-0.05812228	0.57487	-0.0549465
	IL17RB	NM_018725	0.00320971	-1.21518581	0.01276	-0.9942018
		NM_172234	0.00321339	-1.2161327	0.01276	-0.9952967
	IL17RC	NM_153460	0.01095515	-0.41716286	0.05786	-0.2613939
		NM_153461	0.01158635	-0.41533981	0.054	-0.2678711
		NM_032732	0.00998736	-0.42431466	0.05242	-0.2668482
	IL17RD	NM_017563	0.01572808	-0.3488694	0.018	-0.3839925
	IL17RE	NM_153481	0.00346814	-0.70505616	0.00379	-0.7338211
		NM_153480	0.00370663	-0.70220352	0.00379	-0.7303083
		NM_153483	0.00352486	-0.70017927	0.00379	-0.734637
		NM_144640	0.00510833	-0.67756985	0.00379	-0.7237509
IL6	*IL6R*	NM_181359	0.03256818	-0.42177306	0.04104	-0.369827
		NM_000565	0.03400864	-0.41762472	0.04261	-0.3632954

* Positive logFC and q-value < 0.05

Table S3. Pathways that overlapped between expression profiling data from three studies of colonic biopsies and whole blood cells from pediatric ulcerative colitis (z-score > 2 and P value < 0.05)

Positive canonical pathway	Activation z-score			P-value		
	GSE9686	GSE10616	GSE119600	GSE9686	GSE10616	GSE119600
Tec Kinase Signaling	3.81	2.714	3.244	0.002344	0.012882	0.000004
IL-8 Signaling	3.666	3.349	2.885	0.006166	0.011749	0.0002
TREM1 Signaling	3.317	3.651	3.528	0.00003	0.003236	0.000004
Role of Pattern Recognition Receptors in Recognition of Bacteria and Viruses	3.28	2.746	2.6	0.022909	0.012882	0.039811
Production of Nitric Oxide and Reactive Oxygen Species in Macrophages	3.13	3.048	3.464	0.012303	0.00302	0.001072
Leukocyte Extravasation Signaling	2.951	2.63	2.714	0.00000	0.00000	0.012303
Acute Phase Response Signaling	2.898	2.263	3.28	0.006918	0.00138	0.016218
Neuroinflammation Signaling Pathway	2.866	2.36	3.447	0.00912	0.001148	0.013183

Table S4. List of differentially expressed predicted upstream regulators in whole blood cells

Upstream Regulator	Log FC	Activation z-score
VEGF		3.946
HGF		3.674
TGFB1		3.455
OSM	0.406	3.056
AR		2.705
IL6		2.616
EGF		2.525
LIF		2.472
EDNRA		2.449
Nr1h		2.449
INHBA		2.444
FGF2		2.383
26s Proteasome		2.368
CSF1		2.324
CSF3		2.305
PDGF BB		2.282
IGF1		2.247
LRP6		2.216
PGR		2.169
SELP		2.121
FGF8		2.111

Table S5. List of potential biomarkers for ulcerative colitis in whole blood cells

Gene symbol	NCBI Accession	GSE119600 UC (Log FC)
FGFBP2	NM_031950	-0.796
IL32	NM_001012631	-0.686
MUC6	NM_005961	-0.678
LGALS3	NM_001357678	-0.512
PI16[*]	NM_001199159	-0.508
ADM	NM_001124	0.515
PROK2	NM_001126128	0.522
COL18A1	NM_030582	0.539
F5	NM_000130	0.624
JCHAIN	NM_144646	1.09
MZB1[*, #]	NM_016459	1.1
MMP9	NM_004994	1.25

[*]Indicates new potential biomarkers in blood; # Indicates new potential biomarkers in colon.

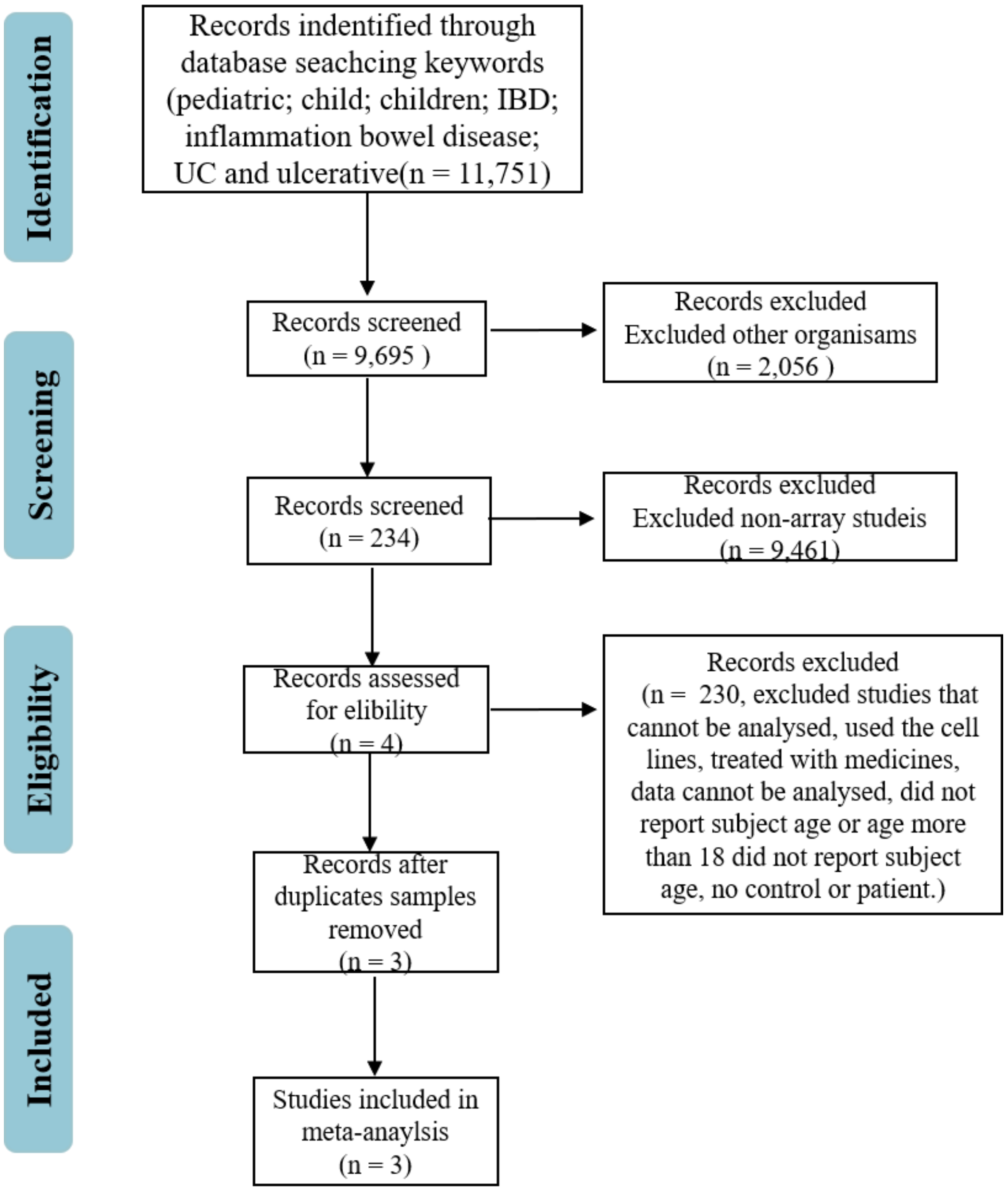

Figure S1. Summary of workflow used for the microarray meta-analysis

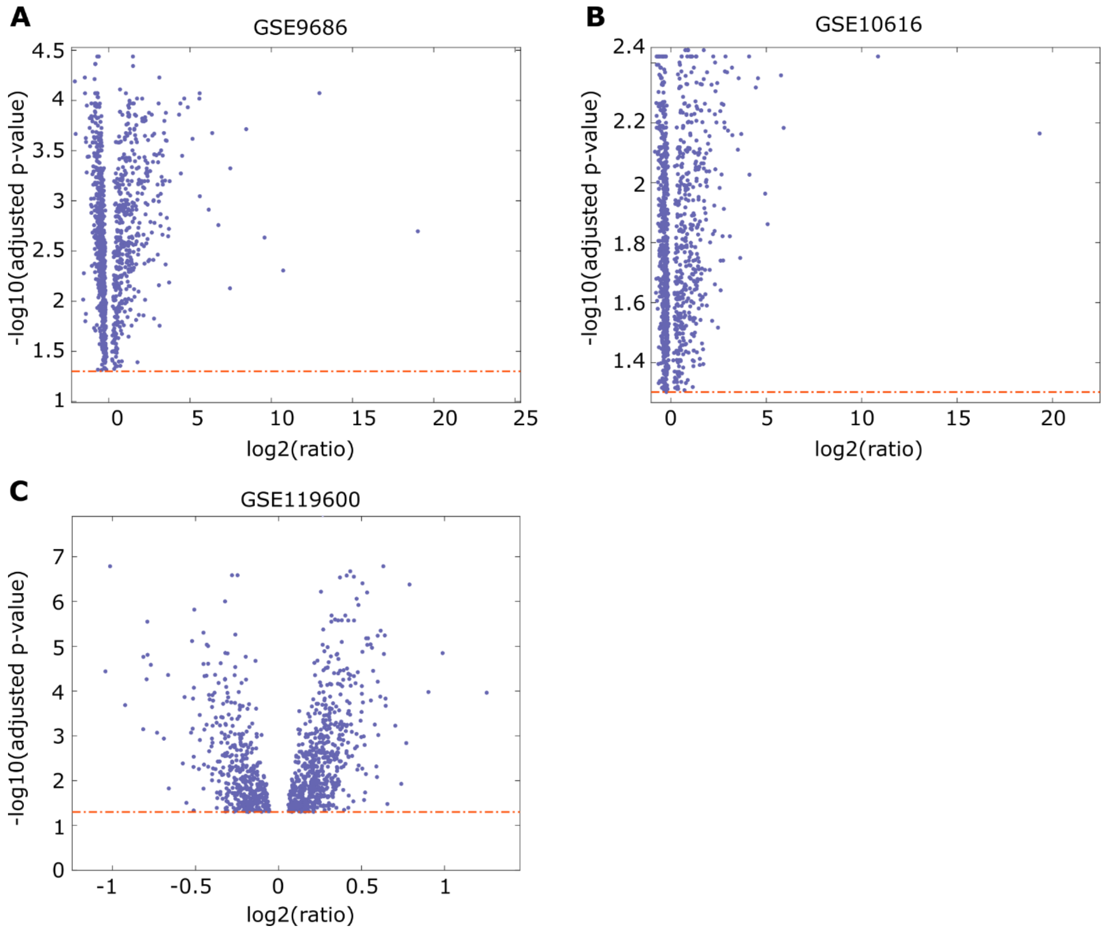

Figure S2. Volcano plots for three datasets

Volcano plots showing differentially expressed genes shared by all datasets. Red horizontal line denotes adjusted P-value cutoff of 0.05
